# Prevalence of sexually transmitted infections, and its associated factors among students in Ethiopia: a systematic review and meta-analysis study

**DOI:** 10.1186/s12889-024-19548-w

**Published:** 2024-07-24

**Authors:** Eyob Ketema Bogale, Tadele Fentabel Anagaw, Misganaw Guadie Tiruneh, Eneyew Talie Fenta, Destaw Endeshaw, Amare Mebrat Delie, Ousman Adal, Abiyu Abadi Tareke

**Affiliations:** 1 Department of Health Promotion and Behavioral Science, School of Public Health, College of Medicine and Health Science, Bahir Dar University, Bahir Dar, Ethiopia; 2 Department of Health Systems and Policy, Institute of Public Health, College of Medicine and Health Sciences, University of Gondar, Gondar, Ethiopia; 3 Department of Public Health, College of Medicine and Health Sciences, Injibara University, Injibara, Ethiopia; 4 Department of Adult Health Nursing, School of Health Sciences, College of Medicine and Health Sciences, Bahir Dar University, Bahir Dar, Ethiopia; 5 Lecturer of Biostatistics, Department of Public Health, College of Medicine and Health Science, Injibara University, Injibara, Ethiopia; 6 College of Medicine and Health Sciences, Department of Emergeny, Bahir Dar University, Bahir Dar, Ethiopia; 7 Amref Health Africa in Ethiopia, COVID-19 Vaccine/EPI Technical Assistant at West Gondar Zonal Health Department, Gondar, Ethiopia

**Keywords:** Meta-analysis, STI, Students, Ethiopia

## Abstract

**Background:**

Sexually transmitted infections (STIs) are illnesses mainly spread through unprotected sexual activity. There is a scarcity of aggregate evidence in Ethiopia. The aim of this review was to assess the pooled prevalence of STI, and its associated factors among students in Ethiopia to fill the aforementioned gap.

**Methods:**

We extensively searched the bibliographic databases of PubMed, Scopus, and Google Scholar to obtain eligible studies. Further screening for a reference list of articles was also done. The Microsoft Excel Spreadsheet was used to extract data, and Stata 17 was used for analysis. The PRISMA-guidline and Newcastle-Ottawa quality assessment scale were used for quality appraisal. To check heterogeneity, the Higgs I2 and Cochran’s Q tests were employed. Sensitivity and subgroup analysis were implemented. To detect publication bias, Egger’s test and funnel plots were used.

**Results:**

The pooled prevalence of STI among students in Ethiopia was 13.6% with a 95% CI (10.2, 17). Findings from sub group analysis based on student category shows that the pooled prevalence of STI were 14.5% among University students, 14.2% among college students and 10.6% among high school students. Having multiple sexual partners (AOR 3.31; 95% CI: 2.40–4.57), not using condoms during sexual intercourse (AOR 2.56; 95% CI: 1.72–3.81), and having poor knowledge about sexually transmitted infections were 3.08 times (AOR 3.08; 95% CI: 1.84–5.15) significantly associated with STI.

**Conclusion:**

The pooled prevalence of STIs among students in Ethiopia was high, and factors like having multiple sexual partners, not using condoms during sexual intercourse, and having poor knowledge about sexually transmitted infections were significantly associated with STIs. Hence, reduce STIs among students, strengthening sexual and reproductive health services, raising awareness about transmission and prevention, and promoting consistent condom use through health information dissemination is crucial. Further qualitative studies are suggested to explore the barriers and facilitators of STI prevention.

## Introduction

Sexually transmitted infections (STIs) are illnesses mainly spread through unprotected sexual activity [1]. More than 30 different bacteria, viruses, and parasites cause STIs. From these microorganisms, four of them namely, syphilis, gonorrhea, chlamydia, and trichomoniasis can be cured [2].

STIs are diagnosed by laboratory testing and clinical presentation. Laboratory diagnosis is the most accurate technique of diagnosis, but because it requires advanced laboratory facilities and skilled workers who can execute technically difficult operations, it is expensive and impracticable in many contexts [3]. In resource-limited countries including Ethiopia, most laboratory investigations to detect STIs are not feasible, thus the diagnosis is based on a syndromic approach which was recommended by WHO [3, 4].

STIs are a major public health concern globally and especially among women in resource-limited countries [5]. Even though strategies were applied for decades, the magnitude of STIs is still high [6]. STIs are among the most common communicable diseases worldwide, and their global burden remains high. According to a World Health Organization (WHO) report, an estimated 374 million new infections with one of four curable STIs occur each year [2]. Globally, the magnitude of these infections varies by region and it is relatively high in the African region [7] with a higher proportion in Sub-Saharan Africa [8].

Different factors, including age, marital status, occupation, level of family income, residence, level of education, length of partnership with current partner, the age difference between the woman and her partner, parity, history of abortion, history of stillbirth, history of STI, gestational age, sexual history with a new partner, number of sexual partners, age at first sexual intercourse, perineal hygiene cleaning practice, and condom use affect STI prevalence [9–11]. Because these factors influence social and sexual networks, access to and provision of care, willingness to seek care, and social norms regarding sex and sexuality, they may pose serious barriers to STI prevention [12].

In addition to the physical health problems including pelvic inflammatory disease (PID), infertility, ectopic pregnancy, chronic pelvic pain, cervical cancer, and urethral stricture [13, 14], diagnosis with STI will have a negative psychosocial and economic impact on the patient and their family [7, 15].

Investigating STI among students are crucial because adolescents and young adults are at a higher risk of contracting STIs due to factors such as risky sexual behavior, lack of information, and vulnerability to peer pressure [16]. Educating students about VCT and HIV prevention can lead to early adoption of healthy behaviors, reducing the long-term risk of HIV transmission and other STIs [17]. Students represent the future generation, and instilling knowledge and positive attitudes towards STIs can have a ripple effect on the broader community [16].

In Ethiopia, though evidence regarding the magnitude is limited, available literature indicated a higher prevalence of STI in the country [9, 18]. To prevent and control STIs, Ethiopia has developed national STI prevention, care, and treatment strategies, programs, and guidelines. Since 2001, Ethiopia has used a syndromic approach to STI screening and treatment, which is one of the services provided [3]. Despite this, the evidence from pieces of studies conducted in different geographical locations in Ethiopia has shown that STIs remain public health challenge in the country.

There is no systematic review or meta-analysis report about prevalence of STI among students in Ethiopia. In addition, systematic review and meta-analysis are needed for the results of earlier research that diverge from one another. Determining the pooled prevalence of STI and related variables among Ethiopian students is the aim of this systematic review and meta-analysis study.

## Methods

### Search strategy

This systematic review and meta-analysis study was undertaken following the preferred reporting items for systematic reviews and meta-analyses (PRISMA) guidelines [19]. This systematic review and meta-analysis study was registered in PROSPERO with reference ID CRD42023486676. The search strategy for this review has been done in two ways. The first was an exploration of electronic databases (PubMed, Scopus, and Google Scholar) for the presence of evidence about prevalence of STI and its associated factors among students in Ethiopia. We have used the following headings and keywords for PubMed database searching: prevalence OR magnitude AND “STI” OR “STD” AND “its associated factors” OR “risk factor” OR “determinant” OR “predictors” AND “ among students in Ethiopia”. Google Scholar and Scopus were conducted in line with database-specific searching guidelines using keywords used in PubMed. The second strategy was a manual search for the reference lists of the incorporated studies. We had put 2007–2023 time restrictions on our search for articles.

### Inclusion and exclusion criteria

An article was eligible for inclusion if the outcome investigated should be STI and its associated factors, and the study had been conducted among students from 2007 to 2023 in Ethiopia. An article was eligible for exclusion if the publication of the article in Non-English language.

### Methods for data extraction and quality assessment

The three aforementioned authors (EKB, TFA, and MGT) independently extracted the essential data from the studies included in the final analysis using a standardized data extraction template. A standardized Microsoft Excel spreadsheet was used to enter the data that had been extracted from the included research. To maintain consistency, cross-checking was done. During the extraction process, discrepancies between the extracted data were resolved through logical discussion among the three authors, and the final consensus was approved with the participation of authors ET and DE.

The table includes the author’s name, the research population, the sample size, the publication year, the study area, the study design, and the evaluation tool for STI and its associated factors among students.

Utilising a PRISMA-compliant assessment form, the data from the included studies was collected [19]. The Newcastle-Ottawa quality assessment scale [20] was used to rate the caliber of the research that was considered in the final analysis. This scale allows for possible scores ranging from 0 to 10. On this scale, a score of 8 or more indicated good quality, a score of 3 to 7 indicated moderate quality, and a score of less than this indicated low quality. The quality of the included studies were evaluated by two independent authors (EKB and TF) using the Newcastle-Ottawa quality assessment scale [20]. The third author (ETF) discussed and resolved any disagreements. Out of the fourteen studies that were considered, ten had good quality and four had moderate quality.

### Data synthesis and analysis

In this meta-analysis, we used a random-effects model to calculate the pooled prevalence of STI and its associated factors. STATA 17 was used for this meta-analysis method. Heterogeneity among the selected studies was assessed using Q and I2 statistics. Heterogeneity is absent when the I2 statistical value is zero, and small, moderate, and substantial heterogeneity are, respectively, indicated by I2 values of 25, 50, and 75% [21].

Potential heterogeneity had an impact on our study; therefore, we did a sensitivity analysis to identify its most likely source. In order to assess STI, a subgroup analysis based on sample size, publication year, and study setting had to be completed. Two tests—the ocular funnel plot test and Egger’s regression test—were used to determine whether there was publication bias. *P*-values < 0.05 were used to define statistical significance for analyses.

## Results

### Identifcation of studies

A total of 1154 papers were found during our search for literature utilizing the previously mentioned search techniques. A second search for the reference list of publications that were supported by references also turned up 7 articles, for a total of 1161 articles. Before the screening, a total of 459 articles were excluded due to duplicate records (*n* = 341), records marked as ineligible by automation tools (*n* = 78), and records removed for other reasons (*n* = 40).

**Fig. 1:**
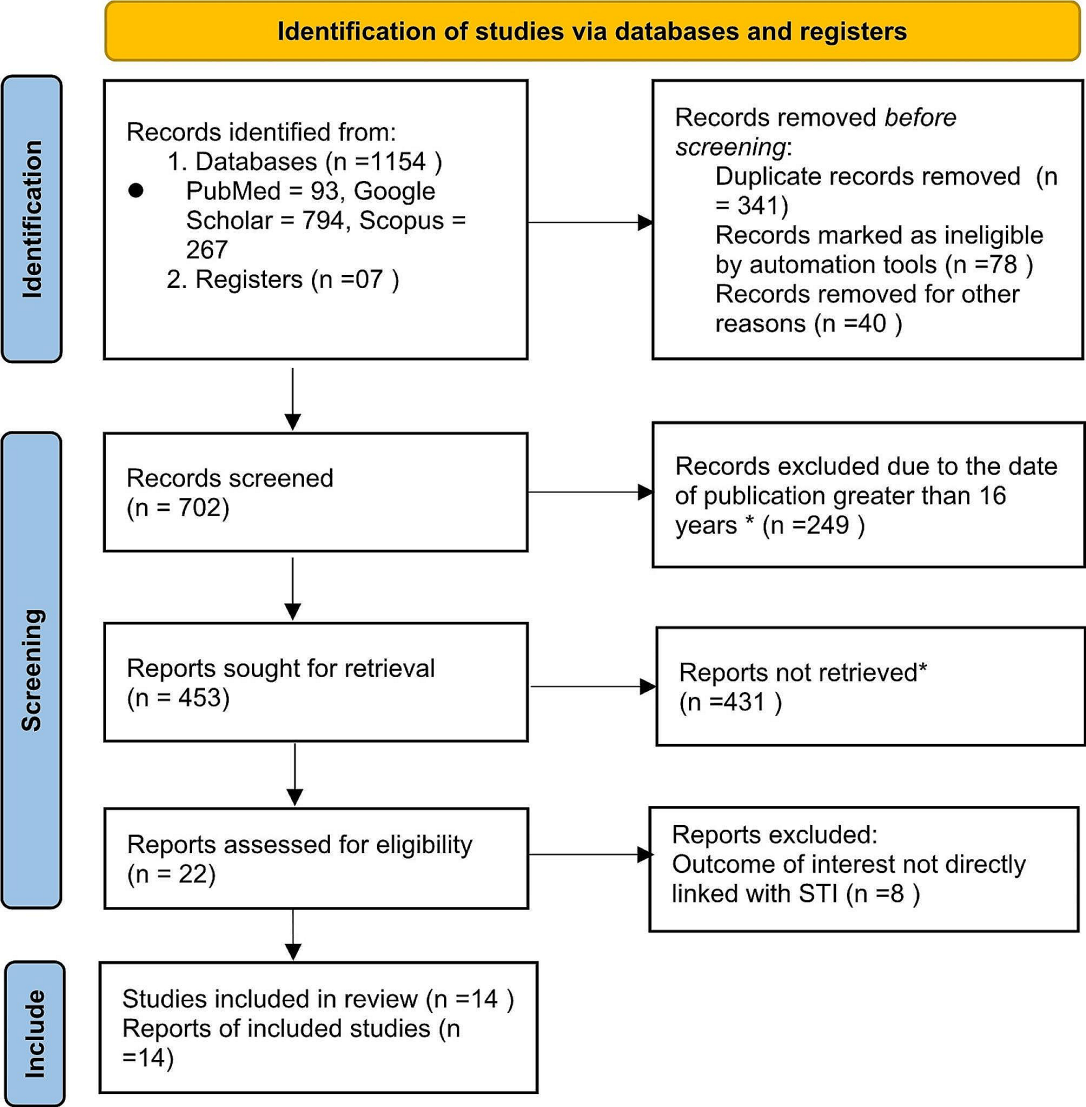
Flow chart for the review process *eliminated by simply reading their titles

During and after screening, a total of 249 of these articles were excluded since their publication dates were more than sixteen years in the past. A total of 431 papers were eliminated by simply reading their titles. The remaining 22 studies were thoroughly examined to see whether they should be included in the meta-analysis; however, only 14 articles [22–35] were included and the remaining 8 articles were also excluded because the outcome of interest does not directly linked with STI (Fig. 1).

### Characteristics of included studies

An overall of 14 studies that examined STI among 7221 students in Ethiopia were included in this systematic review and meta-analysis study [22–35]. Of all the included studies, four were conducted in Oromia [22, 25, 32, 35 ], eight were conducted in Amhara [23, 24, 26, 27, 30, 31, 33, 34], and the remaining two were conducted at SNNPR [28, 29, ]. The study design of all studies was cross-sectional [22–35].

Among the included studies, eight used a sample size of more than 517 [23, 24, 26, 27, 31–34 ], and the remaining six [22, 25, 28–30, 35] used a sample size of less than 517. Moreover, considering the year of publication of the study, seven were published in the past seven years (2017 and above) [22–26, 29, 31], whereas the remaining seven were published between 2007 and 2017 [27, 28, 30, 32–35] (Table 1).

**Table 1:** Characteristics of studies on sexually transmitted infections among students in Ethiopia

Sr. no	Author/year	Study area	Study design	Sample size	Out come variable	% STI
1	Yared A, et al., 2017	Ambo University	CS	400	STI	22.8
2	Gebremichael, H. et al., 2017	High school at Bahir Dar	CS	524	STI	13.1
3	Kassie. BA. et al., 2019	University of Gondar	CS	845	STI	18.2
4	Tamrat, R. et al., 2020	Jimma University	CS	189	STI	14.3
5	Addamu Tobiaw D. et al., 2021	College students at Mota Town	CS	616	STI	14.4
6	Wasie B, et al., 2012	Bahir Dar University and Gondar University	CS	660	STI	6.4
7	Yohannes B, et al., 2013	Wolayita Sodo University	CS	447	STI	19.5
8	Nigusie T, et al., 2020	Highschool students in Mizan Aman Town	CS	360	STI	8
9	Derbie A, et al., 2016	Debre Tabor University	CS	394	STI	12.4
10	Shimie AW, et al., 2022	University of Gondar	CS	844	STI	4.3
11	Sahile Z, et al., 2015	Ambo University	CS	660	STI	6.3
12	Gashaw A, et al., 2007	Gondar Highschool	CS	565	STI	10.7
13	Muluneh M, et al., 2012	Debreberehan University	CS	576	STI	28
14	Alemu ASM, et al., 2015	College students in Bonga	CS	417	STI	13.9

CS: Cross - sectional study

### The pooled prevalence of STI among students in Ethiopia

**Fig. 2:**
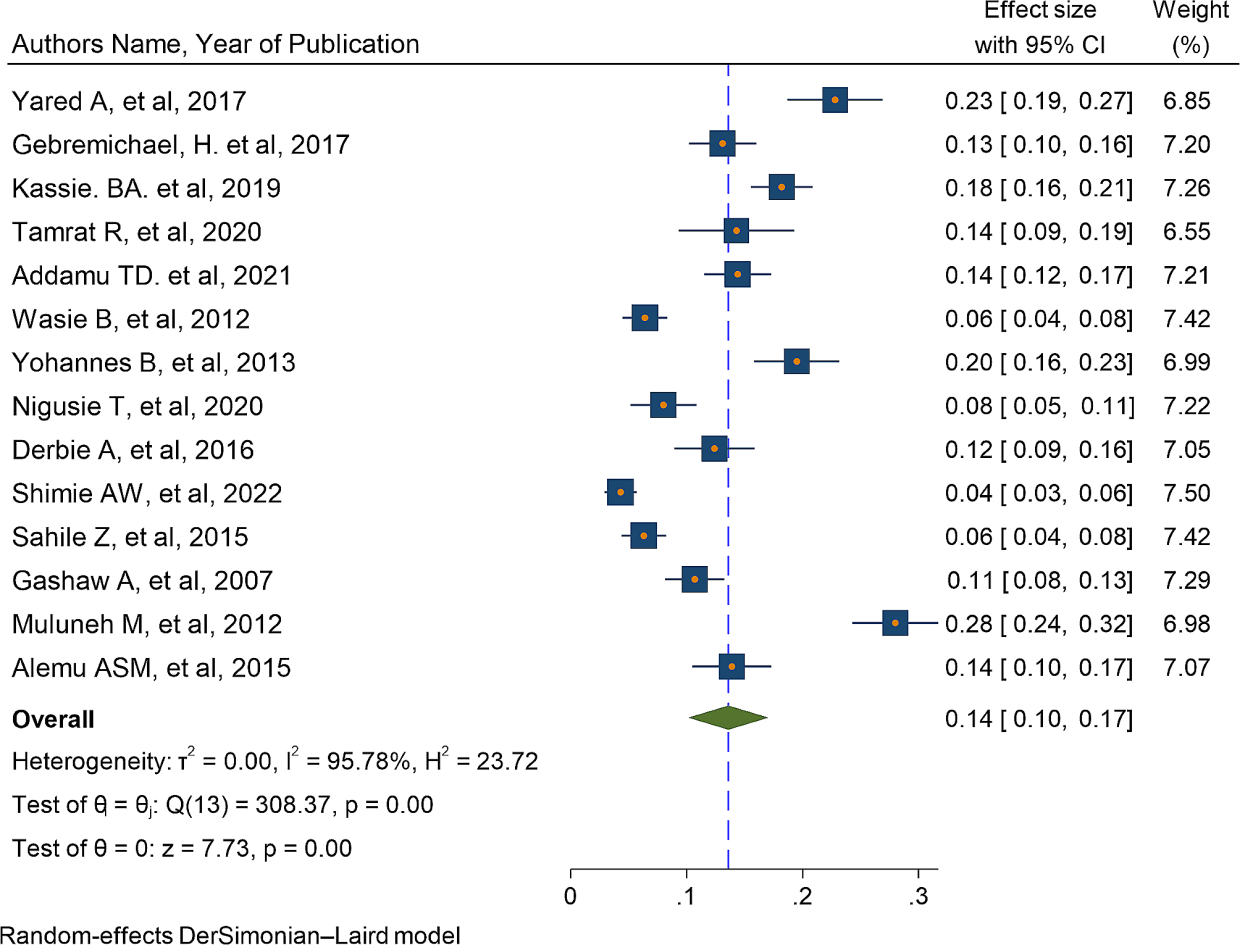
A forest plot for the prevalence of STI among students in Ethiopia

Fourteen studies that assessed STI among students in Ethiopia were included in the final meta-analysis to determine the pooled prevalence of STI. The reported prevalence of STI among the studies that included in the current systematic review and meta-analysis varies from 4.3% in university of Gonder [31] to 22.8% in Ambo University [22]. The pooled prevalence of STI among students in Ethiopia using the random effect model was 13.6% (95% CI 10.2–17). This pooled prevalence was under the influence of significant heterogeneity (I2 = 95.78%, *p*-value < 0.001) from the variance between the included studies (Fig. 2).

### Sub group analysis of the prevalence of STI among adult students in Ethiopia

Since the pooled prevalence of STI was influenced by substantial heterogeneity, a subgroup analysis has been employed based on the study setting where the study was conducted, year of publication, and sample size < or > 517.

The pooled prevalence of STI among students in Oromia region was 14.2% (95% CI 6.7, 21.7), with I2 = 94.90% and a *P* < 0.001. The pooled prevalence of STI among students in Amhara region was 13.3% (95% CI 8.5, 18.2) with I2 = 96.84%, *p*-value < 0.001. The pooled prevalence of STI among students in SNNPR was 13.7% (95% CI 2.4, 25) with I2 = 95.75%, *p*-value < 0.001.

Besides, a subgroup analysis considered the year of publication and sample size. The pooled prevalence of STI among students in Ethiopia was almost similar (13.7% (95% CI 8.6, 18.9) (I2 = 96.5%, *P* < 0.001) in studies published before 2017 [27, 28, 30, 32–35] than in studies published after 2017 [22–26, 29, 31]; 13.5% (95% CI 8.2, 18.7) (I2 = 96.8%, *P* < 0.001).

The pooled prevalence of STI among students was smaller (12.5% (95% CI 8, 17.1) (I2 = 96.94%, *P* < 0.001 in studies that used a sample size ≥ 517 [23, 24, 26, 27, 31–34 ] than in studies that utilized a sample size < 517 [22, 25, 28–30, 35] (15.1% (95% CI 10.6, 19.5) (I2 = 88.66%, *P* < 0.001) [Table 2].

Moreover, The pooled prevalence of STI among University students [22, 24, 25, 27, 28, 30–32, 34 ] was higher (14.5% (95% CI: 9.40, 19.7) (I2 = 97.21%, *P* < 0.001) than in studies [23, 29, 33] among high school students (10.6% (95% CI: 7.80, 13.4) (I2 = 67.03%, *P* = 0.048) and in studies than in studies [26, 25 ] among college students (14.2% (95% CI 12.0, 16.4) (I2 = 0.00%, *P* < 0.868) [Table 2].

**Table 2:** A subgroup analysis of the prevalence of STI among students in Ethiopia

Subgroup	Number of studies	Estimates		Heterogeneity		
		Prevalence (%)	95% CI	I2 (%)	Q(DF)	*P*-value
**Region**						
Amhara	8	13.3	8.5, 18.2	96.84	221.49 (7)	< 0.001
Oromia	4	14.2	6.7, 21.7	94.90	58.77 (3)	< 0.001
SNNPR	2	13.7	2.4, 25	95.75	23.53 (1)	< 0.001
**Sample size**						
< 517	7	15.1	10.6, 19.5	88.66	44.09 (5)	< 0.001
≥ 517	8	12.5	8, 17.1	96.94	228.93 (7)	< 0.001
**Year of publication**						
From 2017–2023	7	13.5	8.2, 18.7	96.8	157.05 (6)	< 0.001
From 2007–2017	7	13.7	8.6, 18.9	96.5	149.61 (6)	< 0.001
**Student Category**						
High school	3	10.6	7.80, 13.4	67.03	6.07 (2)	0.048
College	2	14.2	12.0, 16.4	0.00	0.05 (1)	0.826
University	9	14.5	9.40, 19.7	97.21	286.95 (8)	0.000

CI: confdence interval; DF: degree of freedom;

### Sensitivity analysis

To identify the source of heterogeneity that affects the pooled prevalence of STI among students in Ethiopia, we conducted a sensitivity analysis. According to the findings of the sensitivity analysis, the pooled estimated prevalence of STI obtained when every single study was excluded from the analysis was within the 95% confidence interval of the pooled prevalence of STI when all studies were fitted together. Furthermore, the sensitivity analysis result showed that the pooled prevalence of STI ranges between 4% (95% CI 3, 6) and 28% (95% CI 24, 32) when each study was left out of the analysis (Table 3).

**Table 3:** A sensitivity analysis of the prevalence of STI among students in Ethiopia when each indicated studies are removed at a time with its 95% confdence interval

No	Excluded study	Prevalence of STI (%)	95% CI
1	Yared A, et al., 2017	23	19, 27
2	Gebremichael, H. et al., 2017	13	10, 16
3	Kassie. BA. et al., 2019	18	16, 21
4	Tamrat, R. et al., 2020	14	9, 19
5	Addamu Tobiaw D. et al., 2021	14	12, 17
6	Wasie B, et al., 2012	6	4, 8
7	Yohannes B, et al., 2013	20	16, 23
8	Nigusie T, et al., 2020	8	5, 11
9	Derbie A, et al., 2016	12	9, 16
10	Shimie AW, et al., 2022	4	3, 6
11	Sahile Z, et al., 2015	6	4, 8
12	Gashaw A, et al., 2007	11	8, 13
13	Muluneh M, et al., 2012	28	24, 32
14	Alemu ASM, et al., 2015	14	10, 17

CI: Confdence Interval;

### Publication bias

**Fig. 3:**
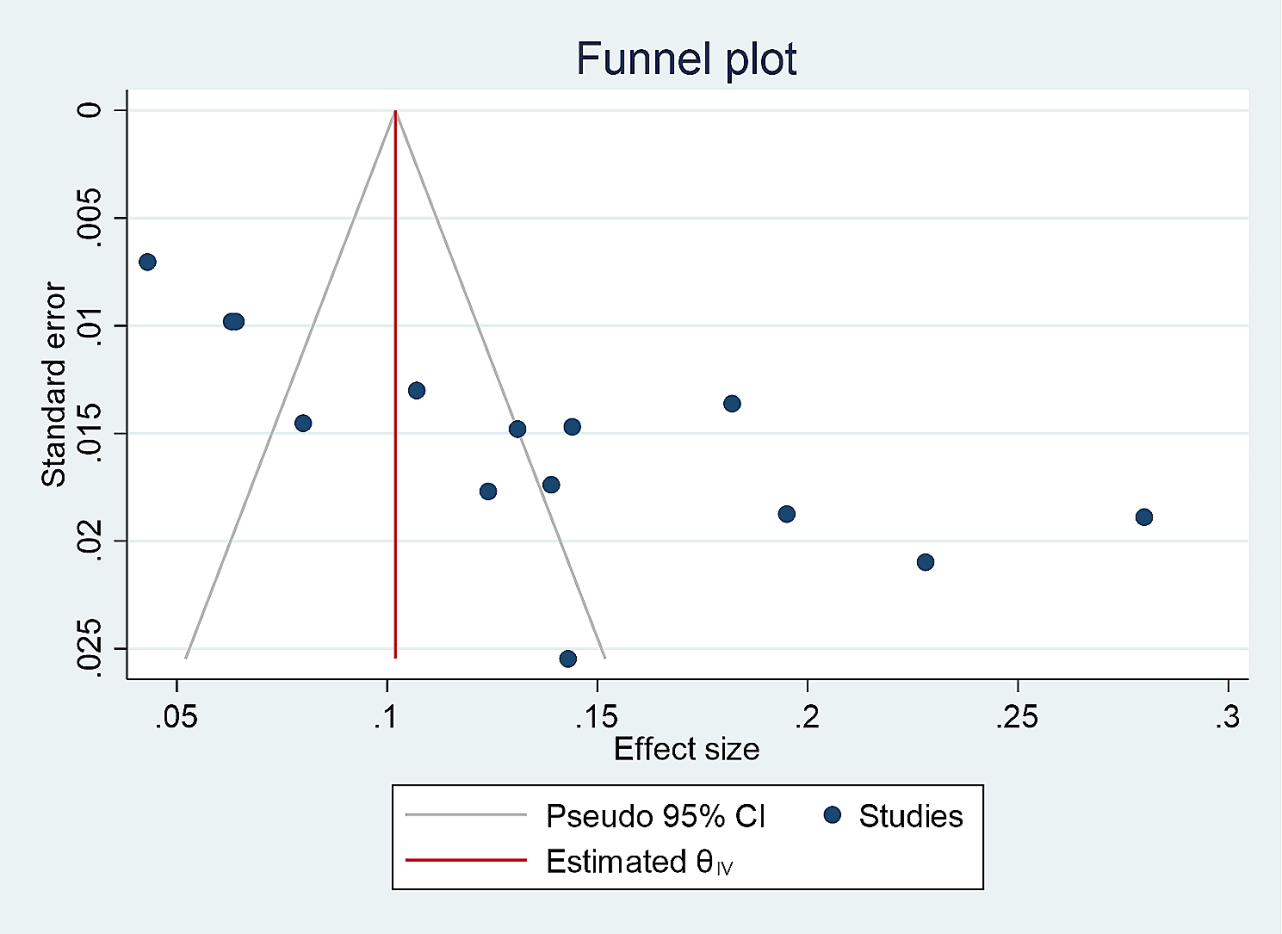
A funnel plot for the prevalence of STI among students in Ethiopia

The presence or absence of publication bias in the prevalence of STI was checked with two methods. The first was Egger’s publication bias plot. The result from this showed that publication bias exists and that its *p*-value is significant (*P*-value = 0.0005) for STI, implying that there is significant publication bias for the prevalence of STI among students in Ethiopia. Moreover, a visual inspection of a funnel plot for a Logit event rate of prevalence of STI among students (Fig. 3) against its standard error suggests supportive evidence for the presence of publication bias. To treat publication bias, we ran a trim-and-fill analysis, however it did not account for additional studies.

### Factors associated with STI among students in Ethiopia

Of all studies, only seven studies reported the factors associated with STI [23, 24, 26]. Having multiple sexual partners [22, 23, 26], not use of condoms during sexual intercourses [24, 26], and poor knowledge of sexually transmitted infections [24, 26] were factors significantly associated with STI.

The pooled effect of three studies [23, 24, 26] showed that students have multiple sexual partners who were 3.31 times (AOR 3.31; 95% CI: 2.40–4.57) more likely to acquire STI in Ethiopia as compared to students who have not multiple sexual partners. The pooled effect of two studies [24, 26] showed that students who did not use condoms during sexual intercourse were 2.56 times (AOR 2.56; 95% CI: 1.72–3.81) more likely to acquire STI in Ethiopia as compared to students who used condoms consistently during sexual intercourse.

The pooled effect of two studies [24, 26] showed that students who had poor knowledge about sexually transmitted infections were 3.08 times (AOR 3.08; 95% CI: 1.84–5.15) more likely to acquire STI in Ethiopia as compared to students who had good knowledge about sexually transmitted infections (Table 4).

**Table 4:** Characteristics of pooled associated factors with STI among students in Ethiopia

Variables	AOR	95% CI	*P* value
Having multiple sexual partners	3.31	2.40–4.57	< 0.001
students who did not use condoms	2.56	1.72–3.81	< 0.001
Poor knowledge about STI	3.08	1.84–5.15	< 0.001

## Discussion

In this systematic review and meta-analysis study, fourteen studies that were conducted between 2007 and 2023 with 7221 participants were analyzed to estimate pooled prevalence of STI, and its associated factors among students in Ethiopia. According to this meta-analysis, the pooled prevalence of STI among students in Ethiopia was 13.6%. Having multiple sexual partners, students who did not use condoms and poor knowledge about STI were significantly associated with STI.

The pooled prevalence of STI among the included studies was 13.6%, with a 95% CI of 10.2–17. This was higher than studies in South African which were 1.5% [36], east Africa, which were 8.4 [36], south east Africa, which was 1.4 [36] and among high school students in Northern Nigeria which was 9.0% [37]. This difference might be due to those studies conducted in Sub Saharan Africa was rely on only laboratory investigation result instead of using syndrome diagnosis. So that laboratory confirmation results less report than syndrome approach.

Having multiple sexual partners [23, 24, 26] was associated with STI among students in Ethiopia. This finding is supported by findings from Ethiopian Federal Ministry of Health [38], Ethiopian Public Health Association [39] and among youth in Malawi [40]. This might be because university and high school students come to a learning center from different areas to access education, they may be vulnerable to have multiple sexual partner due to peer pressure influence and financial incentives from sex seekers. This might be also due to the fact that having multiple sexual partners is one of the risky sexual behaviors that can expose individuals for the risk of contracting sexually transmitted infections including HIV/AIDS [41].

Not using condoms during sexual intercourse [24, 26] was associated with STI among students in Ethiopia. A high rate of not using condoms when having sex indicate that high school students had an increased risk for contracting STIs because unprotected sexual intercourse is one of the major risk factors that exposes students to STIs [38].

In this study, poor knowledge about sexually transmitted infections [24, 26] was associated with STI among students in Ethiopia. This might be due to students who had poor knowledge about sexually transmitted infections engaged in early sexual activity and liable to perform unsafe sex. This in turn could result in occurrence of STIs including HIV/AIDS [42].

### Strengths and limitations of the study

There are strengths and limitations to this systematic review and meta-analysis study. The employment of a predetermined search method that reduces the reviewer’s bias is the first strength of the study. The study’s quality evaluation and data extraction were carried out by independent reviewers, which further reduced reviewer bias. This was the study’s second strength. A strength was the use of sensitivity analysis and subgroup analysis to find the source of heterogeneity. Contrarily, the heterogeneity in the study that could skew the results’ interpretation is what gives birth to its limitations. Another drawback is that the validity of the estimate may be reduced by the subgroup analysis using only a small number of studies. Further more, this study included some studies that are more than 10 years old and pooled prevalence might be biased if estimates has progress over time.

## Conclusion

This systematic review and meta-analysis study revealed that the pooled prevalence of STI among STI among students in Ethiopia was high. Having multiple sexual partners, not using condoms during sexual in intercourse and poor knowledge about STI were significantly associated with STI. Hence, to reduce STI among students, much consideration has to be given to modifying the associated factors by developing and strengthening sexual and reproductive health service provision in high-school and University, and by raising students awareness about STI modes of transmission, and prevention including consistent condom use through health information dissemination. Further qualitative studies are suggested to explore barrier and facilitator of STI prevention.

### Implications of the results for practice, policy, and future research

The results of this systematic review and meta-analysis on STI among students have potential implications for practitioner, policy maker, and future researcher.

**For practitioner**: The finding of this study would be important evidence for clinical practitioners working on STI at ASRH service or youth friendly service to give much emphasis on information dissemination and counseling about STI modes of transmission, prevention including consistent condom use and risky sexual behavior including consequence of having multiple sexual partner, besides to their provision of clinical care.

**For policy Maker**: The policymakers and program planners would design strategies to increase knowledge of students about STI, encourage consistent condom use and discourage risky sexual behavior including having multiple sexual partner among sexually active students.

**For future researcher**: The high pooled prevalence of STI among students will be a motive for further qualitative research to explore barrier and facilitator of STI prevention among students in Ethiopia.

## Data Availability

No datasets were generated or analysed during the current study.

## Abbreviations

AIDS: Acquired Immune-Defciency Syndrome
AOR: Adjusted Odd Ratio CI: Confdence Interval
DF: Degree of Freedom
HIV: Human Immune Virus, STI: Sexually Transmitted Infections

## References

[1] WHO O. Sexually transmitted infections (STIs). Fact Sheets. 2019.

[2] Sexually transmitted infections (STIs). 2019 [cited March, 2023]. https://www.who.int/news-room/fact-sheets/detail/sexually-transmitted-infections-(stis)

[3] FMOH Ethiopia. National Guidelines for the management of sexually transmitted infections using the syndromic approach, 2015.

[4] Workowski KA, Bolan GA. Sexually transmitted diseases treatment guidelines, 2015. Morbidity and mortality weekly report: recommendations and reports. 2015;64(3):1-137. PMC5885289 26042815

[5] Costa MC, Bornhausen Demarch E, Azulay DR, Périssé AR, Dias MF, Nery JA. Sexually transmitted diseases during pregnancy: a synthesis of particularities. Anais brasileiros de dermatologia. 2010;85(6):767–82; quiz 83 – 5. PubMed PMID: 21308300. Epub 2011/02/11. engpor. 10.1590/s0365-05962010000600002 21308300

[6] Grant JS, Chico RM, Lee AC, Low N, Medina-Marino A, Molina RL, et al. Sexually transmitted infections in pregnancy: a narrative review of the global research gaps, challenges, and opportunities. Sex Transm Dis. 2020;47(12):779–89. PubMed PMID: 32773611. Pubmed Central PMCID: PMC7668326. Epub 2020/08/11. eng. 32773611 10.1097/OLQ.0000000000001258 PMC7668326

[7] Organization WH. Report on global sexually transmitted infection surveillance 2018. World Health Organization; 2018.

[8] Ngobese B, Abbai NS. Sexually transmitted infections in pregnant women from Sub-Saharan Africa. South Afr J Infect Dis. 2021;36(1). 10.4102/sajid.v36i1.312 PMC8664065 34917679

[9] Tsega NT, Abebe B, Ebabu T, Asmare T, Kassa M, Haile TT et al. Sexually transmitted infections and associated factors during pregnancy in Gondar city, Northwest Ethiopia, 2021: a multicenter study. Clin Epidemiol Global Health. 2022;16:101096.

[10] Yeganeh N, Kreitchmann R, Leng M, Nielsen-Saines K, Gorbach PM, Klausner J. High prevalence of sexually transmitted infections in pregnant women living in Southern Brazil. Sex Transm Dis. 2021;48(2):128–33. PubMed PMID: 32976355. Pubmed Central PMCID: PMC7817184. Epub 2020/09/26. eng. 32976355 10.1097/OLQ.0000000000001276 PMC7817184

[11] Zango SH, Lingani M, Valea I, Samadoulougou OS, Bihoun B, Rouamba T, et al. Malaria and curable sexually transmitted infections in pregnant women: a two-years observational study in rural Burkina Faso. PLoS ONE. 2020;15(11):e0242368. 33196665 10.1371/journal.pone.0242368 PMC7668607

[12] Institute of Medicine (US) Committee on Prevention and Control of Sexually Transmitted Diseases; Eng TR, Butler WT, editors. The hidden epidemic: confronting sexually transmitted diseases. Washington (DC): National Academies Press (US). 1997. 3, Factors that Contribute to the Hidden Epidemic. https://www.ncbi.nlm.nih.gov/books/NBK232936/25121325

[13] Tsevat DG, Wiesenfeld HC, Parks C, Peipert JF. Sexually transmitted diseases and infertility. Am J Obstet Gynecol. 2017;216(1):1–9. 28007229 10.1016/j.ajog.2016.08.008 PMC5193130

[14] Gomez GB, Kamb ML, Newman LM, Mark J, Broutet N, Hawkes SJ. Untreated maternal syphilis and adverse outcomes of pregnancy: a systematic review and meta-analysis. Bull World Health Organ. 2013;91:217–26. 23476094 10.2471/BLT.12.107623 PMC3590617

[15] Gottlieb SL, Low N, Newman LM, Bolan G, Kamb M, Broutet N. Toward global prevention of sexually transmitted infections (STIs): The need for STI vaccines. Vaccine. 2014;32(14):1527-35. 10.1016/j.vaccine.2013.07.087 PMC6794147 24581979

[16] Mcharo RD, Olomi W, Mayaud P, Msuya SE. Risky sexual behaviours among young adults attending higher learning institutions in Mbeya, Tanzania: implications for STIs and HIV preventive programs. AAS Open Res. 2021;3:41. 10.12688/aasopenres.13123.2. PMID: 37168604; PMCID: PMC10080207. 37168604 10.12688/aasopenres.13123.2 PMC10080207

[17] WHO. Sexually transmitted infections (STIs): key facts. Who. 2016;1–10. Reference Source [Google Scholar].

[18] Kassa D, Gebremichael G, Tilahun T, Ayalkebet A, Abrha Y, Mesfin G, et al. Prevalence of sexually transmitted infections (HIV, hepatitis B virus, herpes simplex virus type 2, and syphilis) in pregnant women in Ethiopia: trends over 10 years (2005–2014). Int J Infect Dis. 2019;79:50–7. 30472433 10.1016/j.ijid.2018.11.009

[19] Moher D, Shamseer L, Clarke M, Ghersi D, Liberati A, Petticrew M, et al. Preferred reporting items for systematic review and meta-analysis protocols (PRISMA-P) 2015 statement. Syst Rev. 2015;4(1):1. 25554246 10.1186/2046-4053-4-1 PMC4320440

[20] Stang A. Critical evaluation of the Newcastle-Ottawa scale for the assessment of the quality of nonrandomized studies in meta-analyses. Eur J Epidemiol. 2010;25(9):603–5. 20652370 10.1007/s10654-010-9491-z

[21] Nyaga VN, Arbyn M, Aerts M. Metaprop: a Stata command to perform meta-analysis of binomial data. Archives Public Health. 2014;72(1):39. 10.1186/2049-3258-72-39 PMC4373114 25810908

[22] Yared A, Sahile Z, Mekuria M. Sexual and reproductive health experience, knowledge and problems among university students in Ambo, central Ethiopia. Reprod Health. 2017;14:41. 10.1186/s12978-017-0302-9 28292296 10.1186/s12978-017-0302-9 PMC5351050

[23] Gebrekidan HG, worku WT, Gebreselassie MA, Yebyo HG, Nigussi DN. Predictors of self-reported sexually transmitted infections among school youth in Bahir-Dar. Magnitude and Northwest Ethiopia. Ethiopian Medical Journal. 2017;55(2). Retrieved from https://emjema.org/index.php/EMJ/article/view/558

[24] Kassie BA, Yenus H, Berhe R, Kassahun EA. Prevalence of sexually transmitted infections and associated factors among the University of Gondar students, Northwest Ethiopia: a cross-sectional study. Reprod Health. 2019;16(1):163. 10.1186/s12978-019-0815-5. PMID: 31703688; PMCID: PMC6842222. 31703688 10.1186/s12978-019-0815-5 PMC6842222

[25] Tamrat R, Kasa T, Sahilemariam Z, Gashaw M. Prevalence and factors associated with sexually transmitted infections among Jimma University students, Southwest Ethiopia. Int J Microbiol. 2020.

[26] Tobiaw D, Muhie O, Reta M. Prevalence and associated factors of self-reported sexually transmitted diseases among college students in Motta Town, Northwest Ethiopia. J Midwifery Reproductive Health. 2021;9(4):2943–51. 10.22038/jmrh.2021.53153.1654 10.22038/jmrh.2021.53153.1654

[27] Wasie B, Belyhun Y, Moges B, Amare B. Effect of emergency oral contraceptive use on condom utilization and sexual risk taking behaviors among university students, Northwest Ethiopia: a cross-sectional study. BMC 137 Res Notes. 2012;5:501. 10.1186/1756-0500-5-501 PMC3494538 22971668

[28] Yohannes B, Gelibo T, Tarekegn M. Prevalence and associated factors of sexually transmitted infections among students of Wolaita Sodo University, Southern Ethiopia. Int J Sci Technol Res. 2013;2(2):86–94.

[29] Nigussie T, Legesse T, Abebe L, Getachew S, Alemayehu D. Magnitude of risky sexual behaviors, determinants, and consequences among high school and preparatory school students in Mizan Aman Town, Ethiopia. J Midwifery Reproductive Health. 2020;8(1):2096–104. 10.22038/jmrh.2019.40248.1450 10.22038/jmrh.2019.40248.1450

[30] Derbie A, Assefa M, Mekonnen D, Biadglegne F. Risky sexual behaviour and associated factors among students of Debre Tabor University, Northwest Ethiopia: a cross-sectional study. Ethiop J Health Dev. 2016;30:11–8.

[31] Shimie AW, Gashu KD, Shiferaw AM, Mengiste SA. Information-seeking behavior on sexually transmitted infections and its associated factors among university students in Ethiopia: a cross-sectional study. Reprod Health. 2022;19(1):25. 10.1186/s12978-022-01340-x. PMID: 35093107; PMCID: PMC8800359. 35093107 10.1186/s12978-022-01340-x PMC8800359

[32] .Sahile Z, Mekuria M, Yared A. Comprehensive HIV/AIDS knowledge and sexual behavior among university students in Ambo, central Ethiopia: implication to improve intervention. J Sex Transm Dis. 2015;2015:890202. 26316983 10.1155/2015/890202 PMC4477256

[33] Gashaw A, Afework K, Feleke M, et al. Low prevalence of HIV infection, and knowledge, attitude and practice on HIV/AIDS among high school students in Gondar, Northwest Ethiopia. Ethiop J Health Dev. 2007;21(2):179–82.

[34] Muluneh M, Wagnew M. Predictors of consistent condom use among University students: hierarchical analysis Debre Berhan,Ethiopia. Global J Med Public Health. 2012;1(4):23–8.

[35] Alemu ASM, Gobena T, Abraha H, Temesgen G, Markos Y. Assessment of substance use and risky sexual behaviour among public college students in Bonga town, Southwest Ethiopia. Am J Biomed Life Sci. 2015;3(5):91–7. 10.11648/j.ajbls.20150305.11

[36] Torrone EA, Morrison CS, Chen P-L, Kwok C, Francis SC, Hayes RJ, et al. Prevalence of sexually transmitted infections and bacterial vaginosis among women in sub-saharan Africa: an individual participant data meta-analysis of 18 HIV prevention studies. PLoS Med. 2018;15(2):e1002511. 10.1371/journal.pmed.1002511 29485986 10.1371/journal.pmed.1002511 PMC5828349

[37] Adeokun LA, Ricketts OL, Ajuwon AJ, Ladipo OA. Sexual and reproductive health knowledge, behavior and education needs of in-school adolescents in Northern Nigeria. Afr J Reprod Health. 2009;13(4):37–49. 20690272

[38] Ethiopian Federal Ministry of Health. National adolescent and youth reproductive health strategy 2007–2015. Available at: countryoffice.unfpa.org/filemanager/files/Ethiopia/ayrh_strategy.pdf

[39] Ethiopian Public Health Association (EPHA). Young people’s HIV/AIDS & reproductive health needs and utilization of services in selected regions of Ethiopia. EPHA; December 2005.

[40] Sathiyasuman A. Associated risk factors of STIs and multiple sexual relationships among youths in Malawi. PLoS ONE. 2015;6(10):8. 10.1371/journal.pone.0134286 PMC4527764 26248328

[41] Centres for Disease Control and Prevention. Youth risk behaviour surveillance-United Sates. 2010.

[42] Inthavong K, Ha LTH, Anh LTK, Sychareun V. Knowledge of safe sex and sexually transmitted infections among high school students, Vientiane Prefecture, Lao PDR. Glob Health Action. 2020;13(sup2):1785159. PMID: 32741352; PMCID: PMC7480502. 32741352 10.1080/16549716.2020.1785159 PMC7480502

